# Climate Change and the Macroeconomic Structure in Pre-Industrial Europe: New Evidence from Wavelet Analysis

**DOI:** 10.1371/journal.pone.0126480

**Published:** 2015-06-03

**Authors:** Qing Pei, David D. Zhang, Guodong Li, Harry F. Lee

**Affiliations:** 1 Department of Geography and International Centre for China Development Study, University of Hong Kong, Pokfulam Road, Hong Kong SAR, China; 2 Department of Statistics and Actuarial Science, University of Hong Kong, Pokfulam Road, Hong Kong SAR, China; Potsdam Institute for Climate Impact Research, GERMANY

## Abstract

The relationship between climate change and the macroeconomy in pre-industrial Europe has attracted considerable attention in recent years. This study follows the combined paradigms of evolutionary economics and ecological economics, in which wavelet analysis (spectrum analysis and coherence analysis) is applied as the first attempt to examine the relationship between climate change and the macroeconomic structure in pre-industrial Europe in the frequency domain. Aside from confirming previous results, this study aims to further substantiate the association between climate change and macroeconomy by presenting new evidence obtained from the wavelet analysis. Our spectrum analysis shows a consistent and continuous frequency band of 60–80 years in the temperature, grain yield ratio, grain price, consumer price index, and real wage throughout the study period. Besides, coherence analysis shows that the macroeconomic structure is shaped more by climate change than population change. In addition, temperature is proven as a key climatic factor that influences the macroeconomic structure. The analysis reveals a unique frequency band of about 20 years (15–35 years) in the temperature in AD1600-1700, which could have contributed to the widespread economic crisis in pre-industrial Europe. Our findings may have indications in re-examining the Malthusian theory.

## Introduction

Climate change has been proven to be the ultimate cause of the large-scale social crisis in pre-industrial Europe [1–3]. Likewise, the prolonged duration of the cooling period in the seventeenth century is regarded as a contributor to the economic crisis [4]. Furthermore, climate change is believed to drive macroeconomic cycles in the long term, which can be confirmed by analyzing low/high-pass filtered data [5]. For these reasons, scrutiny of the relationship between climate change and the macroeconomic structure in pre-industrial Europe in the frequency domain as well as the time domain deserves further attention. Wavelet analysis reveals the characteristics of a data series in the joint frequency and time domains [6, 7]. The relationship between climate change and macroeconomy in pre-industrial Europe can be further investigated based on new findings derived from wavelet analysis, which has been rarely applied to historical/economic geography thus far.

This study aims to be conducted mainly based on the combined paradigms of evolutionary economics and ecological economics. Evolutionary economics allows for economic history to focus on systematic connections and the dynamics of structural change [8]. Furthermore, the effect of the physical environment is regarded as a key in interpreting the causes of economic change [9]. Without considering the environmental factors, major periods and phases in economic history cannot be understood properly under the framework of evolutionary economics [10]. Notably, the study on climate change and the macroeconomic structure of pre-industrial Europe inherently directs to the analysis of ecological economics, which emphasizes the coevolution between an external ecological system and human economies [11]. In fact, ecological economics and evolutionary economics are strongly connected [12] because the former regards development as an evolutionary process with continuous interchange between a changing economy and the environment [13].

Given that disentangling the complex interactions between nature and society is more appropriately set on a large spatial scale [14–16], the study scale is thus set to the entirety of Europe (including all countries), and our study period is delimited to AD1500-1800 to contain relatively rich data. Our chosen time span is nested within the Little Ice Age, which had the lowest temperature throughout the past millennium [17]. Likewise, this period covers the decline of the macroeconomy and the General Crisis of the Seventeenth Century [18].

Although the association between climate change and the macroeconomy in pre-industrial Europe has been revealed in previous studies [4, 5], some essential issues pertinent to the relationship remain unresolved. First, the previous studies were conducted in the time domain; however, quantitative scrutiny in the frequency domain was not carried out. The application of wavelet analysis in this study aims to fill this methodological gap. Second, the economic structure in pre-industrial Europe remains unclear. Wavelet analysis will allow us to capture the evolutionary aspects in the economic structure [19]. Third, the structural changes in climatic conditions during pre-industrial Europe in the frequency domain have not been tested. This study could identify the structural patterns of climate change, which enable a detailed investigation on climatic impact on society, especially during the seventeenth century. In addition, the external impact of climate change could be compared with population pressure, which is a key element in social dynamics. Last, the previous studies used only the 40-year low/high-pass filter in transforming data [20]. Setting 40 years as the criterion for low/high- pass filter has not been verified. This query can be resolved through wavelet analysis.

Wavelet analysis is a powerful tool that addresses the problems of non-stationarity in data series [21]. Given the advantages of wavelet analysis, the macroeconomic structure in pre-industrial Europe and the structural changes affected by climate change could be identified in the frequency domain. In addition to climate change, population dynamics is considered another indispensable internal factor that influenced economic changes in history [22, 23]. Thus, spectrum analysis will evaluate the stability of the macroeconomic structure and coherency analysis will identify whether the macroeconomic structure varied because of climate or population change. The link between climate and social catastrophe in the seventeenth century has been ignored to some extent [24]. Thus, regression analysis in the time domain is employed to further determine the association between climate/population change and the macroeconomy during the General Crisis Period of the Seventeenth Century, which is supplementary to the analysis in the frequency domain.

The datasets are used only to understand macroeconomy under climate change. To achieve our goal, our study is framed on the long-term and continental scale. Therefore, the study focuses neither on individual incidents that can temporarily distort the agrarian economy nor on explanations for several specific cases. Despite its limitations, this broad-brush approach suits the scope of this study to quantitatively identify the relationship between climate change and the macroeconomic structure in pre-industrial Europe at a large spatial and long-term scale.

## Materials and Methodology

**Fig 1:**
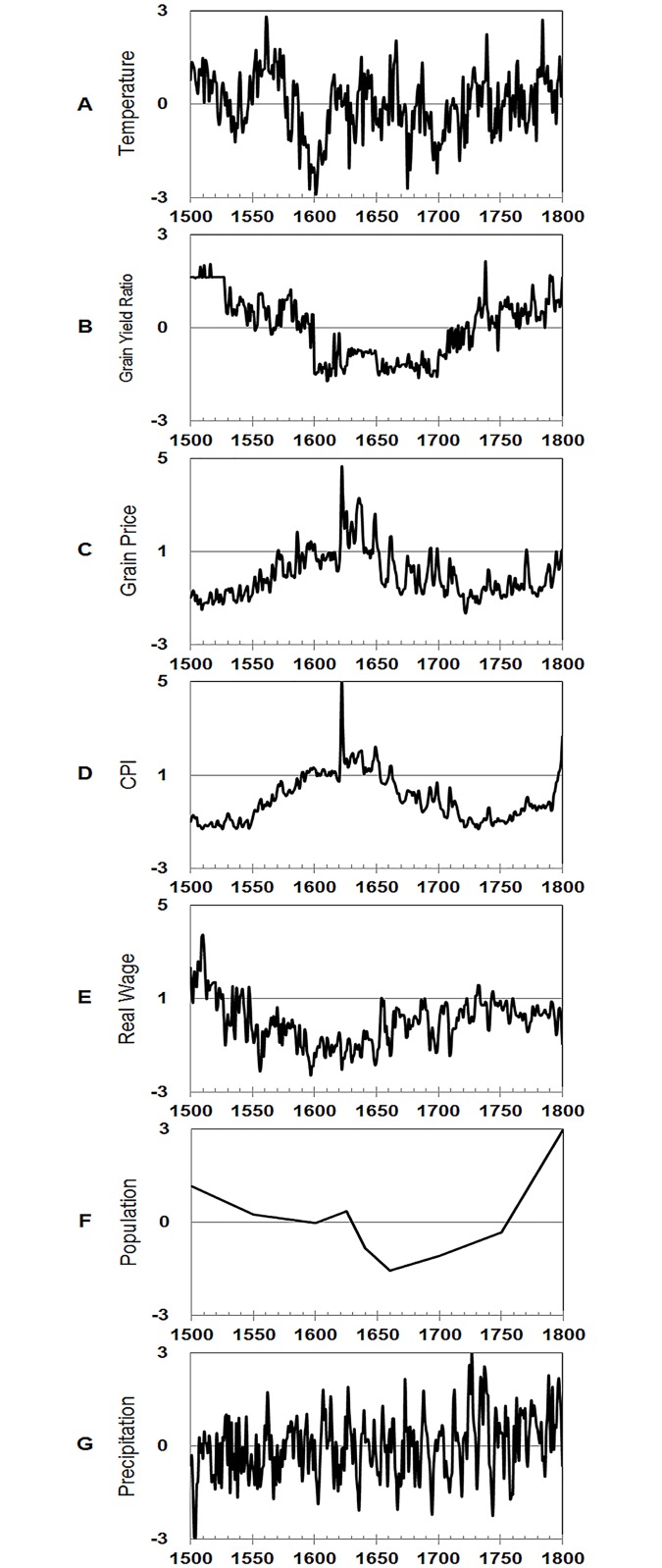
Variables considered in this study. **A**, temperature; **B**, grain yield ratio; **C**, grain price; **D**, CPI; **E**, real wage; **F**, population; and **G**, precipitation. Variables with obvious long-term trends, such as grain price, CPI, real wage, and population were linearly detrended and standardized.

The datasets we used were obtained from previous studies on pre-industrial Europe [1, 4, 5]. These datasets as shown in Fig 1 reflect the general conditions of the macroeconomic structure in the past, through which we could draw quantitative implications using wavelet analysis. In this study, the significance level was set at 0.05 (95%).

### Temperature and Precipitation

Precipitation was excluded in previous studies on climate change and social crisis [1, 25]. However, in the present study, precipitation was included in the analysis to advance the findings regarding the relationship between climate change and society. In this study, anomaly series of temperature and precipitation in Europe were derived from the latest reconstructions based on tree-ring chronologies [26], the data of which have annual resolution. A total of 1,546 tree-ring width series were used for annual temperature reconstruction and 7,284 series for annual precipitation reconstruction. The large sample basis of the data guaranteed the high reliability of paleoclimate reconstruction.

### Grain Yield Ratio (Bio-productivity)

Yield ratio is a useful assessment of economic productivity [27]. In an agrarian society, yield ratio is important in the economy as it reflects the capacity of social production [28]. Our grain yield ratio data were derived from the dataset of van Bath [29], and the grain yield ratio was calculated as follows:

$$\begin{array}{cc} Grain & yield \end{array}=\frac{\begin{array}{cc} Grain & harvest \end{array}}{\begin{array}{cc} Seed & amount \end{array}}$$ (1)

Grain yield ratio referred to the arithmetically averaged yield of four small-grain crops, namely, wheat, rye, barley, and oats. The dataset of van Bath [29] contained the grain yield ratio of 18 pre-industrial countries in Europe and was obtained by arithmetically averaging the grain yield ratios of these countries.

### Grain Price

Our grain price series was derived from data on the website of the International Institute of Social History (http://www.iisg.nl/hpw/data.php#europe). The price dataset covered four types of grains (i.e., wheat, rye, barley, and oats) in 16 major European regions. The unit used was ‘grams of silver per liter’. Grain is a basic human necessity without good substitute, particularly during the European pre-industrial period [30]. The continental integration of the grain market in Europe started within the study period [31, 32]. Thus, this study has a theoretical basis to aggregate prices of grains in Europe. The prices of wheat, rye, barley, and oats were computed to construct the index of grain price series. The annual price series of the four types of grains was arithmetically averaged for use in this study, thereby eliminating noise and attaining an accurate trend.

Nevertheless, although the continental integration of the grain market inside Europe started during the study period [31, 32], the agrarian economy was barely affected by intercontinental trade, considering the high costs of inter-temporal and spatial transport [33]. Therefore, if the economic crisis happened across whole Europe, little could be done to maintain the price level. In addition, although government intervention had previously mitigated recurrent price shocks caused by temporary grain shortages [34], its aid was of minimal help if the scale of the grain shortage was global or spans a large region [2].

### Consumer Price Index

Consumer price index (CPI) measures the cost for an average family to buy a representative basket of goods for daily needs, which include wheat, barley, oats, rye, beef, peas, cheese, eggs, oil, honey, coal, beans, beef, sugar, and butter. CPI is also referred to as the cost-of-living index [35]. The CPI data were from the International Institute of Social History and Allen—Unger Global Commodity Prices Database (http://www.history.ubc.ca/faculty/unger/ECPdb/index.html).

### Real Wage

Real wage pertains to income after taking into consideration the effects of inflation on the purchasing power of the nominal wage. Real wage is regarded as an indicator of previous welfare conditions [36, 37]. Our real wage index was derived from two datasets: the dataset that consists of the real daily wages of farm workers in England [37] and the dataset that comprises the real wages of building artisans and laborers in 19 major European cities, as compiled by Allen [38]. Each real wage series was normalized to homogenize the original variability of all the series. Finally, two normalized series were arithmetically averaged to generate an annual real wage index series.

### Population Size

Although this study does not represent formal demographic research, we also included population as an independent variable in the economic system as recommended in previous studies [1, 5]. Moreover, the increase and decline in population largely influence the economy. In the current study, the European population was obtained from the *Atlas of World Population History* by McEvedy and Jones [39]. On a continental scale, the effects of migration on population were neutralized, because all of Europe is set as the study area.

Population records are incomplete in our study period. There are many ways to handle the missing data, like Singular Spectrum Analysis for the gap filling [40, 41] and the direct wavelet analysis on such unevenly sampled data [42, 43]. However, due to the limited records, we have to follow the conventional practice in interpolating the missing data [44]. Considering that the population data were taken at irregular time intervals, the common logarithm of the data points was determined, then linearly interpolated, and last anti-logged back to create an annual time series. This method prevented any distortion of the population growth rate as a result of the data interpolation. In spite of the coarseness of the population data series, both high- and low-frequency information could be obtained through the application of wavelet analysis. Yet, in the analysis, the limitation of population data should be noted.

Time series with evident long-term trends, such as grain price, CPI, real wage, and population size, was linearly detrended beforehand to extract the real association among the different variables [22], especially for correlation analysis which could be affected by the linear trends [45].

## Analysis and Results

### Correlation Analysis between Climate Change and Macroeconomy

Table 1 shows that the temperature is significantly correlated with all indicators at the significance level of 0.05, but the precipitation is not, which indicates that the precipitation is less important than the temperature in the long term [4, 5]. In any case, population is significantly correlated with all economic indicators at the level of 0.05.

**Table 1:** Correlation results on climate change and macro-economy.

	Grain yield ratio	Grain price	CPI	Real wage	Population size	Precipitation
Temperature	0.356**	-0.169**	-0.197**	0.265**	0.261**	0.045
Grain yield ratio		-0.486**	-0.564**	0.524**	0.672**	0.032
Grain price			0.917**	-0.751**	-0.127*	-0.015
CPI				-0.745**	-0.144*	-0.014
Real wage					0.222**	-0.010
Population size						0.099

Significance:

*p < 0.05, and

**p < 0.01.

### Wavelet Analysis of Climate Change and Macroeconomy

Wavelet analysis is a powerful tool that has already been applied in science and engineering. The application of wavelet analysis in the social sciences is still limited. In the study, we follow the Cazelles’s practice [7] to implement wavelet analysis to investigate the relationship.

#### Choice of the Mother Wavelet

Wavelet transform decomposes signals using dilated and translated functions called “mother wavelets.” In this study, we selected Morlet wavelet as the mother wavelet, which is well localized in scales and in high-frequency resolution when compared with Mexican hat [7]. Morlet wavelet is regarded as an efficient means of detecting and analyzing curves [46]. In the context of ecological study, the Morlet wavelet and Mexican hat came to similar conclusions concerning the features of the wavelets [47]. Therefore, because of the abovementioned technical advantages, the Morlet wavelet has been selected as the mother wavelet in the analysis. In the meantime, such selection of the mother wavelet in our study has also considered other application practices of wavelet, like Mexican hat.

#### Spectrum Analysis of Climate Change and Macroeconomy

**Fig 2:**
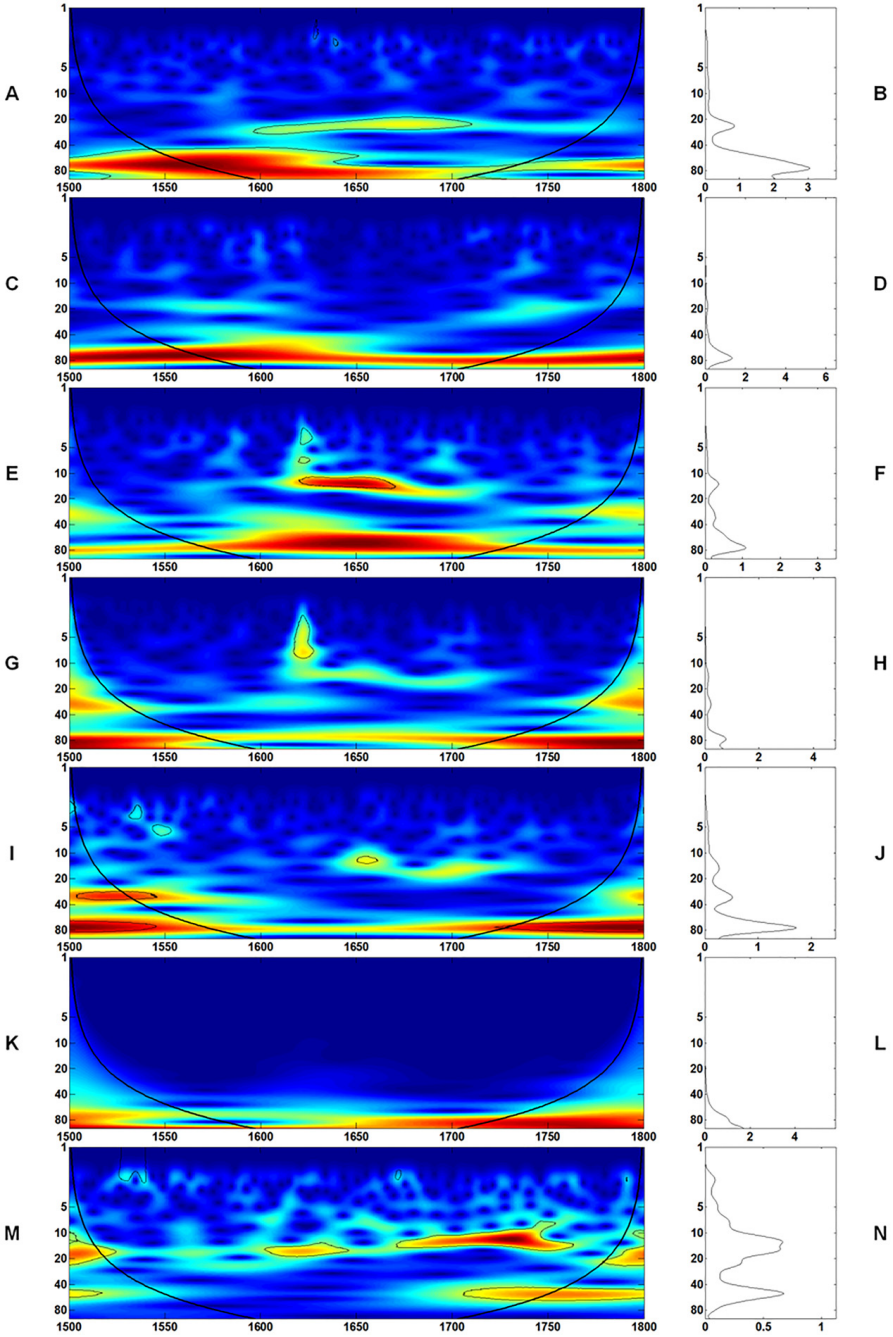
The continuous wavelet power spectrum (left panels) and average wavelet power spectrum (right panels, solid lines) in 1- to 100-year band of (A, B) temperature in AD 1500–AD 1800; (C, D) grain yield ratio in AD 1500–AD 1800; (E, F) grain price in AD 1500–AD 1800; (G, H) CPI in AD 1500–AD 1800; (I, J) real wage in AD 1500–AD 1800; (K, L) population in AD 1500–AD 1800, and (M, N) precipitation in AD 1500–AD 1800 in Europe. The color code for coherence values transitions from dark blue (low values) to dark red (high values). The black line delimits the region that is not influenced by edge effects.

Spectrum analysis is useful for identifying the informative features of many types of real-world signals [48]. Fig 2 shows wavelet spectrum results from 1 to 100 years. Setting the upper limit to larger than 100 years is unreliable, as it results in fewer than three cycles in the entire study period. As indicated in Fig 2, the frequency patterns of each data series, except for precipitation, have a stable 60–80 year frequency band. This result is consistent with the previous correlation analysis and further proves that the amount of rainfall is less important to affect agrarian economy [4, 5].

Consequently, the roles of temperature and population in an agrarian economy merit further analysis. As shown in Fig 2, in all the indicators, frequency of less than 10 years hardly exists. Therefore, in the coherence analysis in Section 3.2.3, only temperature and population size are considered in the test pairs, namely, temperature—yield, temperature—price, temperature—CPI, temperature—real wage, population—yield, population—price, population—CPI, and population—real wage, at the frequency band of 10–100 years. At the same time, the band of 60–80 years in particular is compared. In the meantime, there is a differentiated band of 80-year and above in population. Therefore, the coherency analysis will further compare the relationship between temperature/population changes with other indicators.

#### Coherence Analysis of Climate Change and Macroeconomy

**Fig 3:**
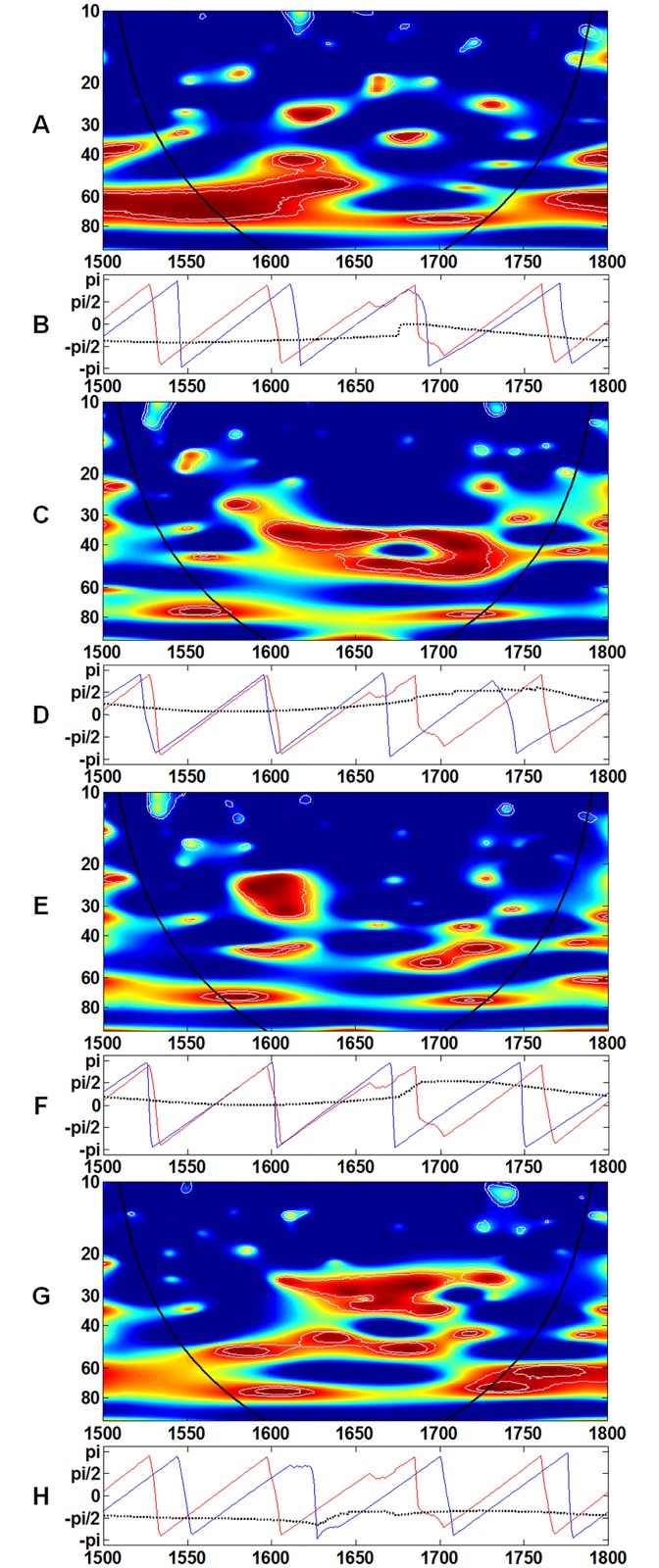
Wavelet coherence between A, temperature and yield; C, temperature and price; E, temperature and CPI; and G, temperature and real wage is computed in the 10- to 100-year band. The color code for coherence values changes from dark blue (low values) to dark red (high values). The phases for **B**, temperature and yield; **D**, temperature and price; **F**, temperature and CPI; and **H**, temperature and real wage are computed in the 60- to 80-year band. The dotted line represents phase difference; the red line represents the phase of temperature; and the blue line represents the phase of the corresponding variable. The black line delimits the region that is not influenced by edge effects.

**Fig 4:**
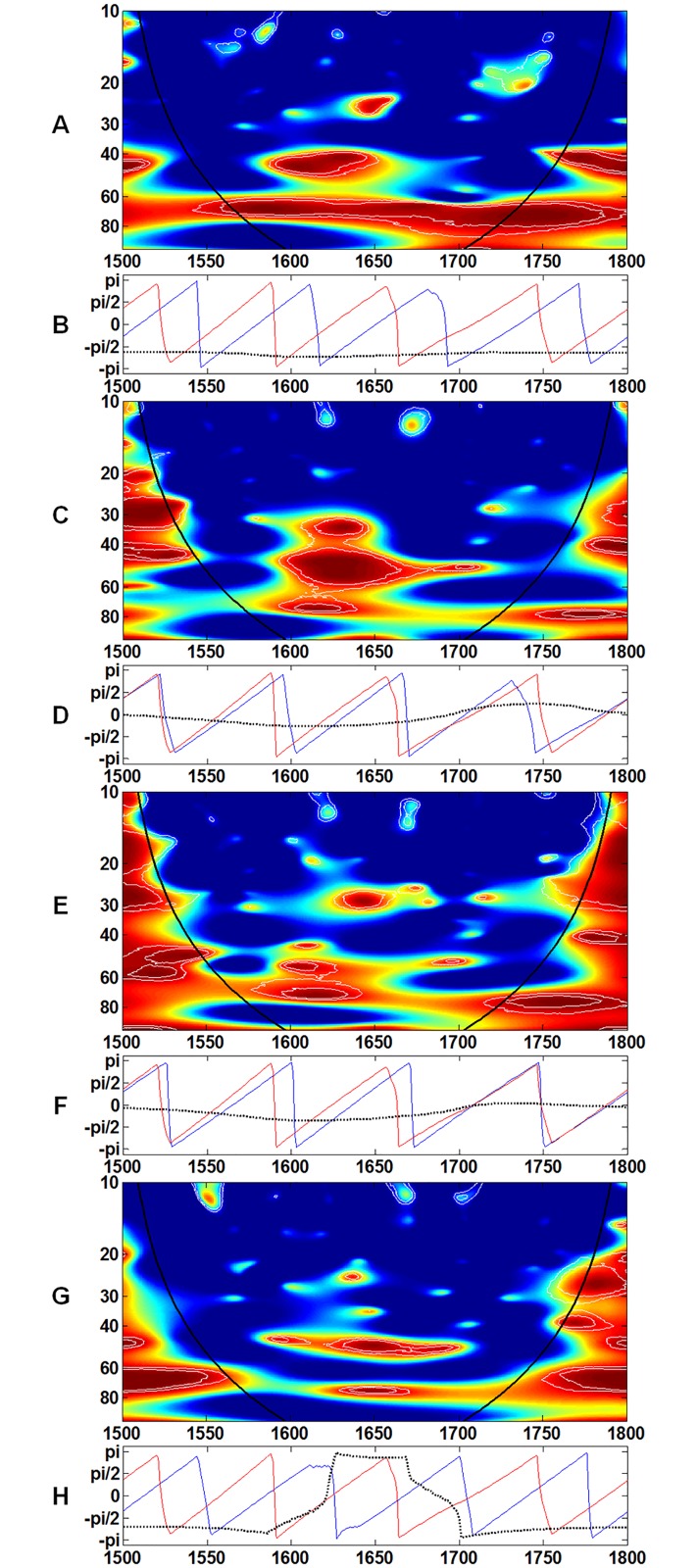
Wavelet coherence between A, population and yield; C, population and price; E, population and CPI; and G, population and real wage is computed in the 10- to 100-year band. The color code for coherence values changes from dark blue (low values) to dark red (high values). The phases for **B**, population and yield; **D**, population and price; **F**, population and CPI; **H**, population and real wage are computed in the 60- to 80-year band. The dotted line represents phase difference; the red line represents the phase of population; and the blue line represents the phase of the corresponding variable. The black line delimits the region that is not influenced by edge effects.

Coherence is a direct measure of the association between two time series in the frequency domain [49]. Fig 3 shows the results of the coherence analysis of test pairs with temperature. Each pair shows a stable and coherent result, particularly at the 60–80 year band (Fig 3B, 3D, 3F and 3H). Fig 4 shows each test pair with population. Unlike the test pair of temperature and real wage, the test pair of population and real wage is not stable at the 60–80 year band (Fig 4H). For the other three pairs, the results are relatively steady at the 60–80 year band (Fig 4B, 4D and 4F). The band of 80-year and above in population could not affect these economic indicators either.

## Discussion

### Structural Identifications of Macroeconomy

Structural changes in the economic system are one of the key research interests of evolutionary economics, which has a firm empirical basis [50, 51]. In many occasions, we may reasonably ask whether a trend changes in every period or whether it changes only occasionally [52]. Hence, identifying the structure of an agrarian economy in the past can help us understand the economic fluctuations during the study period.

First, a consistent and continuous frequency band of 60–80 years was observed, as shown in Fig 2. This frequency band is obvious not only for the temperature (Fig 2A and 2B) but also for other series of indicators (Fig 2C–2L). This frequency band is stable throughout the entire study period, which may show that real linkages exist among these indicators, particularly at the long-term band of 60–80 years. Since the results of wavelet analysis could only justify the stability of the macroeconomic structure at the continental scale, we cannot comment on the occurrence of structural changes in any specific region or a limited short-term period.

Second, grain yield ratio has been considered an effective indicator of past agricultural production efficiency and technology, particularly during the period when grain, a basic necessity of humans, has few known substitutes [30]. An impression of the vulnerability of the surpluses, on which the complex civilization of Europe relied, can be obtained by examining harvest yield ratios [53]. However, the frequency domain analysis results on yield ratio indicate that throughout the study period, hardly any structural changes occurred in agricultural technology. These results provide a quantitative justification for several current ideas that little change occurred in the technological level in agriculture or in the economic structure in pre-industrial Europe [37, 54, 55].

Third, after closely inspecting the results of the spectrum analysis of the seventeenth century, a short frequency band in temperature of around 20 years (15–35 years) is observed. The frequency bands of 15–35 years and 60–80 years are also found in Lüdecke et al’s (2013) study about the long-term changes of European temperature [56]. Those bands also appear in grain price, CPI, and real wage. However, such an anomaly does not occur in grain yield ratio and population. Yield ratio is used to indicate only the productivity in the agricultural sector and is still essentially different from agricultural production. The seventeenth century was a chaotic period in Europe. If the economic crisis is related to the change in population, a similar signal should be observed in the macroeconomy. The emphasis on climate change in triggering crisis has been seriously discussed recently together with overpopulation [24], although statistical evidence is still inadequate, especially in the frequency domain. The relationship between macroeconomy and climate/population change is further investigated using coherence analysis and discussed in the following section.

Fourth, the present study based on wavelet analysis confirms the previous findings [4, 5] about the relatively less importance of precipitation. The spectrum of precipitation (Fig 2M and 2N) is different from those of temperature, population, and four other indicators. No consistent and continuous frequency band of 60–80 years is found for the precipitation. Nevertheless, the importance of precipitation in agriculture at the regional or local level within a short temporal duration cannot be denied [4, 5].

### Macroeconomic Structure under the Impact of Climate/Population Change

In Section 4.1, the frequency band of 60–80 years in temperature, and other indicators has been shown in Fig 2. In this section, the influence of climate/population change on the macroeconomic structure is explored through coherence analysis.

Based on the results of the coherence analysis (Figs 3 and 4), both temperature and population have important contributions to the economic system in the low- frequency (long term) aspect. The most consistent and continuous coherent region (red color in the core area) occurs in the band of 40 years and above. Therefore, the previous studies that adopted 40 years as the smoothing criterion included almost all influences on society from both external (climate change) and internal (population change) factors [1, 2, 4, 5, 20]. However, a smoothing criterion of 40 years has never been verified by analytical studies on climate change and social responses.

Remarkably, the coherence analysis of population and real wage merits further discussion. As shown in Fig 4H, the phase difference in the coherence analysis of population and real wage changes from (–Pi/2) to (Pi/2) in the band of 60–80 years. Test pairs with population and three other factors show the stability in the band of 60–80 years (Fig 4B, 4D and 4F). By contrast, phase difference is stable in the band of 60–80 years in the coherence analysis of temperature and real wage, as well as those of temperature and other indicators (Fig 3B, 3D, 3F and 3H). Hence, if grain yield ratio, grain price, CPI, and real wage are considered to represent the economic mechanism in pre-industrial Europe, the coherence analysis indicates that such mechanism is shaped more closely and stably by climate change than population change.

In addition, during the seventeenth century, the spectrums of grain price, CPI, and real wage (Fig 2E, 2G and 2I) are marked by the existence of the 15–35 year frequency band, which is similar to that of temperature. Based on the results of the coherence analysis, the highly coherent region with population primarily exists for 40 years and above in AD1600-1700 (Fig 4A, 4C, 4E and 4G). The highly coherent region below 40 years occurs only in population—price (AD1600-1700) (Fig 4C) and population—CPI (AD1620-1660) (Fig 4E). Compared with population, the highly coherent region below 40 years occurs in almost all the test pairs with temperature (Fig 3A, 3C, 3E and 3G). The highly coherent region with temperature for price and real wage could reach 20–30 years in AD1600-1700 (Fig 3C). In particular, for temperature—CPI, a highly coherent region occurs in the band of 20–30 years from AD1600 to 1630 (Fig 3E) when CPI rises sharply, as shown in Fig 1D. Overall, in the seventeenth century, the severe climatic cooling is associated with a unique frequency band in the wavelet analysis, which could ultimately distort the structure of other economic indicators.

The influence of climate change on the economy and society of pre-industrial Europe from AD1500 to 1800 in the time domain has been discussed in previous studies [1, 5]. Therefore, we specifically initiate the regression analysis of temperature, population, and four other indicators only in the seventeenth century, instead of covering the entire study period. According to Figs 2 and 3, the unique frequency band of 20 years (15 to 35 years) in the temperature has been revealed. Therefore, to retain most signals, 15-year low-pass filter is used to smooth temperature and four other indicators. However, a 40-year low-pass filter is still retained for population and four other indicators. As the length of the examined period is only one hundred years, only regression analysis of temperature and economic mechanism is carried out to confirm whether climate/population change exerts any influence on the economy (Tables 2 and 3). To address autoregressive disturbances in the economic time series of grain price, CPI, and real wage, Prais—Winsten estimation is adopted to address the flexible time trend (*t*, *t* ^2^, and *t* ^3^) [4, 25]. For grain yield ratio, the regression is directly implemented with temperature and population because of their instantaneous association, especially in the pre-industrial era.

**Table 2:** Regression results on temperature change and macro-economy by 15 year low-pass filtered data.

	Dependents			
	Grain Yield Ratio	Grain Price	CPI	Real Wage
**Constant**	3.904**	-0.016	0.100**	-0.275**
**Temperature**	0.161**	-0.024*	-0.025**	0.169**
***t***	---	0.012**	0.015**	-0.033**
***t*** ^***2***^	---	-1.950E-04**	-2.360E-04**	0.001**
***t*** ^***3***^	---	7.953E-013**	8.094E-013**	-2.919E-012**

Significance:

*p < 0.05, and

**p < 0.01.

**Table 3:** Regression results on population change and macro-economy by 40 year low-pass filtered data.

	Dependents			
	Grain Yield Ratio	Grain Price	CPI	Real Wage
**Constant**	3.806**	0.046**	0.187**	-0.751**
**Population**	0.006	0.007**	-0.001	-0.005
***t***	---	0.010**	0.011**	-0.011**
***t*** ^***2***^	---	-1.410E-04**	-1.890E-04**	3.160E-04**
***t*** ^***3***^	---	5.204E-013**	6.510E-013**	-1.462E-012**

Significance:

*p < 0.05, and

**p < 0.01.

As shown in Table 2, all coefficients of temperature are significant at the 0.05 level using 15-year low-pass filtered data. Such results confirm that temperature influences the economy in pre-industrial Europe as the identified frequency band in Figs 2 and 3. The sudden occurrence of the 15–35 years frequency in the temperature deserves further investigation. However, the results in Table 3 indicate that the coefficients of population in the regression with grain yield and real wage are not significant at the 0.05 level. This indicates that the effect of population on pre-industrial European economy was not as significant as that of climate change during AD1600 to 1700.

The general interpretations of the processes of long-term economic change in late medieval and early modern Europe usually pertain to demographic fluctuations and the growth of trade and markets [57]. However, this study provides statistical evidence to re-examine the role of population pressure in triggering the economic crisis. Notably, Malthusian theory and Darwin’s theory, among others, focused only on the cumulative population pressure that led to the socio-economic crisis and ignored the impact of climate change. However, the current study has identified that climate change has a more significant role than population pressure during the seventeenth century (see Tables 2 and 3). Aside from demographic impact, the rise of capitalism also seems to have transformed the social and economic structure in agrarian society, particularly in the long run [58]. Although many other socio-economic factors were adopted to account for this period of crisis, climate change, particularly cooling, should not be neglected in the analysis of the seventeenth-century crisis in Europe. Based on the analysis in the frequency domain, this study proposes the first time that the frequency band of 15 to 35 years in the temperature has induced unexpected effects on the macroeconomic structure in pre-industrial Europe.

Contemporary historians have depicted a systemic “General Crisis,” typified by economic distress, political unrest, and population decline in Europe in the seventeenth century. The time span of the General Crisis of the Seventeenth Century in Europe is generally considered from AD1590 to 1660 [59], whereas some scholars believe that the crisis began in AD1560 when hyperinflation started [60]. This period is also called “Little Ice Age” with severe cooling by climate historians, as it destroyed the agricultural production in Europe [61]. Based on research in paleo-climate change, the cooling occurred all over Europe and decreased the continent’s bio-productivity dramatically [26, 62, 63].

As a consequence, local failures in agrarian economy were frequent and led to high market prices because of drastic food shortages during this period [64]. The European economy in the seventeenth century totally collapsed. The successive harvest failures across Europe imposed economic distress to the political situation [65]. In addition to the investigation on the macro-scale, country-level studies also show that the cooling caused the shrinkage of harvest and led to miserable conditions. For instance, England experienced several frequent high-mortality famines because of low agricultural production [66]. An economic recession occurred in Germany because of the bad harvest during the Little Ice Age [67]. The Thirty Years’ War even accelerated the deterioration of the German economy [68]. Many French historians have commented that large sections of France suffered repeated famines, which resulted in inadequate food sources in the seventeenth century [69]. In Eastern Europe, Poland and Russia did not escape famines [70, 71]. As agricultural shrinkage could also lead to a general decline in nutrition, diseases are normally endemic among the population and could suddenly increase in virulence and rampancy [72].

In summary, the harvest failure under climate change led to the sharp rise in prices, the effect of which spread widely from country to country, from class to class, and even from individual to individual [73]. Although different reasons (wars, famines, and epidemics, among others) for the chaos exist, most European countries suffered significantly during the same time span. Climate change has been proven to be the fundamental reason for the continent-wide crisis in pre-industrial Europe [1–3]. The above phenomena further justify the significance of this research on the relationship between climate change and the agrarian economy in pre-industrial societies [24].

## Conclusions

We applied wavelet analysis as the first attempt to investigate the relationship between climate change and macroeconomic structure in pre-industrial Europe in the frequency domain. Our results show that there was a consistent and continuous frequency band of 60–80 years in the temperature, and four other factors during the study period. In addition, we have reaffirmed that temperature is the major climatic factor that influences economic structures. Results of the wavelet analysis show that an economic structure is primarily affected by the change in temperature. The analysis has detected the first time a frequency band of 15 to 35 years in the temperature in AD1600-1700, which significantly contributed to the widespread economic crisis in pre-industrial Europe. Finally, the results of the analysis on grain yield ratio in the frequency domain show that the technological level in pre-industrial Europe remained stagnated.

This study does not refute other theories on agrarian European macroeconomy on other temporal and spatial scales, and does not lead to a conclusion of environmental determinism. In line with the integrated paradigms of evolutionary economics and ecological economics, the study simply aims to provide new evidence from wavelet analysis in the frequency domain, which has never been studied before in terms of research on the relationship between climate change and macroeconomic structure at the continental level in pre-industrial Europe. The introduction of the new method, namely, wavelet analysis in the frequency domain, can help us explore new results on the traditional subject of the climate—economy interaction in human society.
